# Correlation between Reduced FEF25-75% and a Positive Methacholine Challenge Test in Adults with Nonobstructive Baseline Spirometry

**DOI:** 10.1155/2021/6959322

**Published:** 2021-12-29

**Authors:** Irfan Shafiq, Mateen Haider Uzbeck, Zaid Zoumot, Mohamed Abuzakouk, Niyas Parappurath, Ali Saeed Wahla

**Affiliations:** Respiratory and Allergy Institute, Cleveland Clinic Abu Dhabi, Abu Dhabi, UAE

## Abstract

**Objective:**

To clarify whether in adults with a nonobstructive spirometry a reduced FEF25-75% is associated with a positive methacholine challenge test (MCT).

**Methods:**

Data was collected for all the patients who had a MCT done between April 2014 and January 2020 but had nonobstructive baseline spirometry. Logistic regression was utilized to estimate the log odds of a positive methacholine test as a function of FEF25-75% and also for age, gender, BMI, FEV1, and FEV1/FVC.

**Results:**

Out of 496 patients, 187 (38%) had a positive MCT. Baseline characteristics in two groups were similar except that patients with positive MCT were younger (32 ± 11.57 vs. 38 ± 13.25 years, respectively, *p* < 0.001). Mean FEF25-75% was lower in MCT positive (3.12 ± 0.99 L/s) vs. MCT negative (3.39 ± 0.97 L/s) patients, *p* = 0.003. Logistic regression results suggest that MCT outcome is inversely related to FEF25-75%, age, and gender. Specifically, as FEF25-75% percentage of predicted value increases, the log odds of a positive MCT decrease (odds ratio (OR) = 0.90, 95% confidence intervals (CI) = 0.84‐0.96, *p* = 0.002). Also, as age increases, the log odds of a positive MCT decrease (OR = 0.95, 95%CI = 0.94‐0.97, *p* < 0.001).

**Conclusions:**

Reduced FEF25-75% in adults with nonobstructive spirometry can predict a positive response to MCT in younger patients. However, this relationship becomes weaker with increasing age.

## 1. Introduction

Asthma remains one of the most prevalent chronic respiratory illnesses and carries a high clinical and socioeconomic burden [1]. An accurate diagnosis of asthma is of paramount importance as it can avoid unnecessary investigations and costly treatments for patients [2]. However, at the milder end of the spectrum, the diagnosis can be difficult to establish as the baseline spirometry in this group of patients is often normal while the symptoms are in remission. Airway hyperresponsiveness is a key component of bronchial asthma [3], and demonstrating its presence or absence can greatly help in establishing a diagnosis and guiding treatment. Bronchial challenge tests are commonly utilized in the secondary and tertiary care setting to look for airway hyperresponsiveness and can be performed using inhaled methacholine, histamine, and mannitol or by exercise or eucapnic hyperventilation as provocative stimulus. These tests have high sensitivity but low specificity for diagnosis of asthma [4, 5]. The methacholine challenge test (MCT) is one of the commonest tests used in clinical practice to elicit airway hyperresponsiveness and is the test we use at our institution.

The majority of mild asthma patients are managed in primary care facilities where bronchial challenge testing is not readily available, and simple spirometry is the only diagnostic tool at hand. This raises the question of whether other spirometry-derived parameters can help identify patients who could have airway hyperresponsiveness and would benefit from either further testing to confirm the diagnosis of asthma, or in whom a trial of treatment with inhaled corticosteroids is warranted.

Forced expiratory volume in 1 second (FEV1), forced vital capacity (FVC), and FEV1/FVC are the usual measurements of interest in patients with obstructive airway disease. Forced expiratory flow 25–75 (FEF25-75%) is also routinely measured and reported on spirometry. It is defined as “forced expiratory flow over the middle one-half of the FVC; the average flow from the point at which 25% of the FVC has been exhaled to the point at which 75% of the FVC has been exhaled.” A reduced FEF25-75% is thought to be a marker of small airway obstruction [6], and there is now ample evidence of small airway obstruction in asthma [2, 7]. FEF25-75% has been reported to be reduced in children with asthma [8], associated with increased asthma severity in adults [9], and also correlates well with both a raised fractional exhaled nitric oxide (FeNO) and a raised peripheral blood eosinophil count [10]. Some authors have suggested that FEF25-75% can be used as an early marker of airway hyperreactivity in mild asthma [11]; however, its clinical significance especially in adults without a prior diagnosis of asthma and a nonobstructive spirogram remains unclear.

Our study is aimed at investigating whether a reduced FEF25-75% is associated with increased airway hyperresponsiveness as demonstrated by a positive methacholine challenge test, in adults with asthma-like symptoms and a nonobstructive spirogram (Normal FEV1 and FEV1/FVC).

## 2. Methods

### 2.1. Subjects

In this retrospective observational study, we identified and reviewed the electronic medical records of all adult patients who underwent a methacholine challenge test between April 2014 and January 2020, having presented to our respiratory clinic with asthma-like symptoms (i.e., a chronic cough, SOB, or intermittent wheeze), and had at least one baseline spirometry assessment which ruled out airflow obstruction. Patients with a prior confirmed diagnosis of asthma and those with alternate diagnoses that could explain their symptoms were excluded from the study.

### 2.2. Study Variables

We recorded absolute values as well as % predicted values for FEV1, FEV1/FVC, and FEF25-75% for all participants. Only the tests that met the American Thoracic Society (ATS) criteria for spirometry were included in the study, and Global Lung Function Initiative (GLI) reference values were used to determine the % predicted values. The results of methacholine challenge results were recorded as positive or negative. As per the ATS guidelines [5], a PC20 (provocative concentration causing a 20% fall in FEV1) of more than 16 mg/mL was recorded as a negative MCT. Demographic data including gender, age, and BMI was also collected for all participants.

### 2.3. Statistical Analysis

Logistic regression was utilized to estimate the log odds of a positive methacholine test as a function of patient age, gender, BMI, FEF25-75%, FEV1, and FEV1/FVC. As a first step in the model building process, main effects, two-way interactions between clinical variables, and 2^nd^ and 3^rd^ degree polynomial terms for continuous variables were all examined. Model reduction was performed in a stepwise fashion by removing interaction and higher-order polynomial terms that failed to reach statistical significant at the 0.05 level. Main effects were retained in the final model in all instances; however, all nonsignificant (*p* > 0.05) main effects were grand-mean centered to facilitate model interpretation. Proportions were used as descriptive statistics for categorical variables, and data are expressed as the mean ± standard deviation (SD). Comparisons of values between groups were performed by using Student's *t*-test, and *p* < 0.05 was considered statistically significant.

## 3. Results

Four hundred and ninety-six patients had a methacholine challenge test between April 2014 and January 2020 and met the inclusion criteria for the study including 186 (37.5%) men and 310 (62.5%) women. MCT was positive in 187 (38%) and negative in 309 (62%) patients. Mean age of the study subjects was 36 ± 12.97 years. Patients with positive MCT were significantly younger, mean age 32 ± 11.57 vs. 38 ± 13.25 years, respectively, *p* < 0.001. There was no significant difference between the two groups in terms of BMI. Mean FEF25-75% was significantly lower in MCT positive (3.12 ± 0.99 L/s) vs. MCT negative (3.39 ± 0.97 L/s) patients, *p* = 0.003. This effect was more pronounced on comparison of FEF25-75% percentage of predicted values, 88.29 ± 22.05% for MCT positive vs. 100.13 ± 22.85% for negative patients, *p* < 0.001. Prebronchodilator FEV1 and FEV1% predicted values were not significantly different between the two patient groups. Baseline characteristics and outcomes are listed in Table 1.

The final logistic regression model along with odds ratio point estimate results is provided in Table 2. Logistic regression results suggest that the MCT outcome is related to patient's FEF25-75%% predicted, age, and gender. Specifically, as FEF25-75% percentage of predicted value increases, the log odds of a positive MCT decrease (OR = 0.90, 95%CI = 0.84‐0.96, *p* = 0.002), though the associated 2^nd^ degree polynomial term indicates that the rate of this decrease lessens as FEF25-75% predicted values increase (coef = 0.0003, OR = 1, *p* = 0.028). Similarly, as age increases, the log odds of a positive MCT also decrease (OR = 0.95, 95%CI = 0.94‐0.97, *p* < 0.001). Finally, we found that males have lower odds of a positive MCT relative to females (OR = 0.56, 95%CI = 0.37‐0.85, *p* = 0.006).

**Figure 1:**
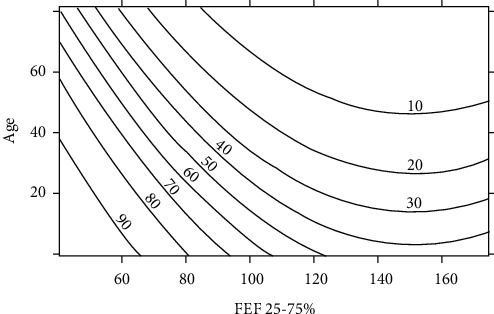
Probability of positive methacholine test contour graph: age by FEF% predicted value.

Next, the estimated log odds based on age and FEF25-75%% predicted values were transformed into probabilities. A contour graph was then constructed to show how the probability of a positive MCT changes as a function of age and FEF25-75% percentage of predicted values—see Figure 1. Here, the *x*-axis and *y*-axis represent FEF25-75% percentage of predicted value and age, respectively, while the contour lines represent the probability of a positive test.

**Figure 2:**
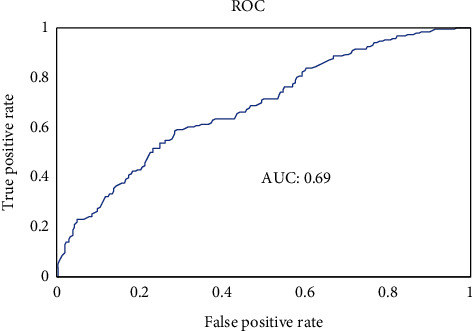
Receiver operating characteristic curve.

The Receiver Operating Characteristic (ROC) curve resulting from the final model is shown in Figure 2. Overall, the area under the ROC curve is approximately 0.69 suggesting that over the full range of probability thresholds for classification, the model has low to moderate predictive value.

## 4. Discussion

Our study shows reduced midexpiratory flow rates as evidenced by a low FEF25-75% in patients with nonobstructive spirometry, but asthma-like symptoms can predict a positive response to MCT in younger patients. However, this relationship becomes weaker with increasing age. Hence, it is perhaps unsurprising that the association between FEF25-75% and asthma has been more established in pediatric than the adult populations.

Boggs et al. were one of the earliest groups to look at FEF25-75% in asthmatics [12]. In 1982, they evaluated the clinical significance of FEF25-75% among 167 known asthmatics and its utility in predicting airway responsiveness to inhaled bronchodilators and noted that FEF25-75% provided no benefit over FEV1 in predicting response to bronchodilation. Subsequently, in the 2005 American Thoracic Society (ATS) guidelines [13], it was observed that although the earliest change associated with airflow obstruction is a reduction in midflow (FEF25-75%) and terminal flow; however, these midflow measurements are not specific for small airway disease in individual patients.

The Severe Asthma Research Program (SARP) looked at 829 severe adult asthmatics and noted that FEF25-75% was an independent predictor of severe asthma symptoms after controlling for FEV1, FEV1/FVC, and demographic variables [14]. Their results were similar to those of Rao and colleagues who looked at 744 children diagnosed with asthma and noted that among those patients who had a normal FEV1, a reduced FEF25-75% was associated with increased asthma severity, exacerbations, and need for systemic steroids [15].

Malerba et al. looked at the association between impairment in FEF25-75% and bronchial hyperresponsiveness through methacholine challenge testing in adults [11]. They followed 400 consecutive patients aged 17.8-41.4 years who had normal FEV1, FVC, and FEV1/FVC but presented with asthma-like symptoms; FEF25-75% was abnormal in 27.5% of these patients. Two hundred and twenty-four had a positive methacholine challenge test (defined as PD_20_ < 16 mg/mL). A significant moderate positive correlation between bronchial hyperresponsiveness and FEF25-75% (Spearman's coefficient 0.339, *p* < 0.001) was noted. However, the authors did not look at differences in bronchial hyperresponsiveness based upon the age of patients. Additionally, patients with lower FEF25-75% were noted to have higher fractional exhaled nitric oxide (FeNO) levels and sputum eosinophils. In a separate study, Raji and colleagues also looked at FEF25-75% values in 234 patients with normal FEV1 and FEV1/FVC ratios with asthma-like symptoms who went for bronchial hyperresponsiveness testing [16]. They observed that mean FEF25-75% was 70.9 ± 19.2% for patients with a positive methacholine test versus 84.2 ± 22.7% for those with a negative methacholine test (*p* < 0.001). However, they were unable to determine an optimal discrimination threshold of FEF25-75% that could be used as a cut-off for predicting bronchial hyperresponsiveness. Drewek and colleagues also looked at a pediatric population of 532 children aged 4-18 years and observed that those patients with a positive methacholine challenge test were more likely to have reduced FEF25-75% [17]. They also suggested that FEF25-75% may be used as an adjunct to FEV1 to define a positive methacholine study. Similarly, another study also showed reduced FEF25-75% to be associated with long-term persistence of asthma and risk of poor asthma outcomes, even after adjustment for FEV1 [18].

Most recently, Hou et al. performed a multicenter retrospective cross-sectional study of 846 Chinese adults with suspected asthma and FEV1 ≥ 80% predicted and found that FEF25-75% values were significantly lower and FENO values significantly higher in patients with a positive methacholine challenge test. Combining these two values gave an area under the curve (AUC) of 0.865 (95% CI: 0.833-0.893). They observed that the optimal cut-off for predicting a positive methacholine results to be FEF25-75% of less than 74.9% predicted in patients younger than 55 years of age and less than 62.9% in patients > 55 years of age [19].

FEF25-75% also has certain limitations due to the fact that it is directly dependent on FVC maneuver. Hence, if the FVC is reduced due to the patient effort during the test, then the FEF25-75% will also be reduced. This error can be minimized by making sure that the patient understands the test instructions and that the ATS criteria for spirometry performance are followed and met. Secondly, the FVC and can also be reduced due to a disease process which in turn will affect the FEF25-75%. For this reason, we only included the patients without preexisting lung disease in our study [20].

Our study is unique as it demonstrates a variable relationship between FEF25-75% and a positive MCT depending on the age of the subjects as it shows that younger patients with a reduced FEF25-75% were more likely to have a positive MCT as compared to an older patient with the same degree of midrange airflow limitation. The use of Figure 1 allows a clinician to decide the probability of a positive methacholine tested based upon a patient's age. For example, a 20-year-old patient with FEF25-75% 60% of predicted would have a greater than 90% chance of having a positive methacholine test; however, a 40-year-old patient with the same FEF25-75% would have a greater than 60% chance of a positive methacholine test and this would reduce further to 40% for a 60-year-old patient. Hence, our experience shows that the cut-off threshold for predicting a positive response to bronchial hyperresponsiveness testing varies with the age of the patient.

The limitations of our study include the fact that it is a retrospective one and most of the patients were of a single (Arab) ethnic group; hence, there is a need to validate our age-based threshold cut-off findings in a prospective manner and test these thresholds cut-offs in different ethnicities. Also, since most of the MCTs and the data collection was done prior to the European Respiratory Society's guidance on technical standards for bronchial challenge testing [21], the MCTs were performed and interpreted using PC20 rather than PD20. Lastly, the appropriate cut-offs for defining an abnormal FEF25-75% have not been defined properly especially in adults. A cut-off value of 65% has been suggested as abnormal by Ciprandi et al. [8] based on their observation of this parameter in children with an established diagnosis of asthma; however, the appropriate reference range in adults and in those without a preexisting asthma diagnosis is not really known.

In conclusion, this study shows an age-related correlation between the midrange flow limitation and presence of bronchial hyperresponsiveness in adult patients, meaning that younger patients had a much higher likelihood of a positive MCT if they had reduced FEF25-75% which in turn could be a marker of undiagnosed asthma and a predictor of BHR positivity in a select group of younger patients.

## Figures and Tables

**Table 1:** Baseline characteristics and results.

	Total	Positive	Negative	*p* value
*n*	496	187 (38%)	309 (62%)	
Male	186	58 (31%)	128 (69%)	
Female	310	129 (42%)	181 (58%)	
Age	36 ± 12.97	32 ± 11.57	38 ± 13.25	<0.001
BMI	28.27 ± 6.28	27.76 ± 6.72	28.58 ± 5.99	0.169
FEF25-75% (L/s)	3.29 ± 0.98	3.12 ± 0.99	3.39 ± 0.97	0.003
FEF25-75% (percentage of predicted)	95.67 ± 23.25	88.29 ± 22.05	100.13 ± 22.85	<0.001
FEV1/FVC	83.43 ± 5.91	83.94 ± 6.53	82.59 ± 5.45	0.019
Prebronchodilator FEV1	3.00 ± 0.72	2.96 ± 0.69	3.02 ± 0.74	0.367
FEV1% predicted	90.32 ± 11.14	89.13 ± 11.01	91.04 ± 11.18	0.064

Results shown as *n* (%) and means ± SD.

**Table 2:** Logistic regression results.

Predictors	*p* value	Odds ratio (95% CI)
Age	0.000	0.95 (0.94-0.97)
Male (vs. female)	0.006	0.56 (0.37-0.85)
BMI	0.839	1.00 (0.97-1.04)
FEV1 (% of predicted)	0.169	1.02 (0.99-1.04)
FEF25-75% (% of predicted)	0.002	0.90 (0.84-0.96)

Outcome = methacholine test result; negative = 0; positive =1.

## Data Availability

The data used to support the findings of this study are available from the corresponding author upon request.
